# Comparing Spinal Chloroprocaine to Hyperbaric and Isobaric Bupivacaine for Total Hip and Knee Arthroplasties: A Retrospective Study

**DOI:** 10.7759/cureus.35729

**Published:** 2023-03-03

**Authors:** Lisa Gu, Cameron R Smith, Barys Ihnatsenka, Yury Zasimovich, Linda Le-Wendling

**Affiliations:** 1 Anesthesiology, University of Texas Southwestern, Dallas, USA; 2 Anesthesiology, University of Florida, Gainesville, USA

**Keywords:** spinal chloroprocaine, intrathecal chloroprocaine, postoperative urinary retention, ambulatory surgery, hip total arthroplasty, knee total arthroplasty, chloroprocaine, anesthesia spinal

## Abstract

Introduction: Spinal anesthesia is commonly used for total knee and hip arthroplasties (TKA/THA). The rising popularity of ambulatory TKA and THAs require anesthetic techniques that provide rapid recovery of motor and sensory function while minimizing side effects like postoperative urinary retention. This single-center retrospective observational study compares the recovery profile of patients undergoing TKA and THA under chloroprocaine spinals compared to hyperbaric and isobaric bupivacaine spinals.

Methods: One hundred and twelve patients undergoing primary TKA and THA under spinal anesthesia at University of Florida Health were identified between September 1, 2019 and February 21, 2020. Their electronic medical records were reviewed. Patients were categorized based on the local anesthetic used in the spinal. Various demographic, intraoperative, and postoperative data were compiled and compared, including duration of surgery, time to physical therapy, time to post-anesthesia care unit (PACU) discharge, and time to spontaneous micturition.

Results: Time to spontaneous micturition and PACU discharge were significantly lower in the chloroprocaine spinal group compared to the hyperbaric bupivacaine group by 193 minutes and 42 minutes, respectively. Fewer patients receiving chloroprocaine spinals had their first physical therapy session limited by residual motor weakness compared to those in both bupivacaine groups. Additionally, mean duration of surgery was shorter in the chloroprocaine group compared to both bupivacaine groups (89 minutes compared to 111 minutes). Time to physical therapy completion was not different. All groups had <10% conversion to general anesthesia.

Conclusion: Chloroprocaine spinals can be feasible options for TKAs and THAs with improved postoperative recovery profiles compared to bupivacaine spinals.

## Introduction

The ideal spinal anesthetic provides adequate surgical anesthesia for the entire duration of the surgery, wears off quickly afterwards, and results in minimal side effects during and after the surgery. Bupivacaine is commonly used for spinal anesthesia for total hip arthroplasties (THA) and total knee arthroplasties (TKA). However, residual lower extremity weakness and postoperative urinary retention (POUR) can delay physical therapy and hinder early discharge [1]. Both of these side effects of spinal anesthesia are counterproductive for enhanced recovery after surgery (ERAS) and ambulatory joint surgery.

Partly because of concerns about residual motor weakness and POUR after spinal anesthetics, the use of spinal anesthesia for ambulatory surgeries is being called into question, particularly with “fast-track general anesthesia” becoming more popular [2]. However, if shorter acting local anesthetics can minimize postoperative spinal complications, spinal anesthesia can still be a viable option for ambulatory TKA and THAs. While the improvement of general anesthetics over the last decades have decreased the clear benefits of spinal anesthesia over general anesthesia for lower extremity joint replacements, patients who undergo spinal anesthesia for same day TKA and THA may still be more likely discharged successfully on the same day [3].

Chloroprocaine spinals had fallen out of favor in the 1980s after multiple cases of neurologic injury were observed following inadvertent intrathecal injections of 3% 2-chloroprocaine during intended epidural delivery [4-5]. A new formulation of 1% chloroprocaine was approved by the USA Food and Drug Administration for intrathecal use in 2017 with improved safety profile. Intrathecal chloroprocaine result in reliably shorter duration motor and sensory blockade and faster time to micturition and ambulation when compared to intrathecal bupivacaine [6-7]. For primary TKAs and THAs, chloroprocaine spinals may provide a better recovery profile compared to bupivacaine spinal anesthesia, provided that surgical anesthesia duration is adequate.

Since this FDA-approved formulation of intrathecal chloroprocaine became available at the authors’ institution, anesthesiologists at this hospital system began to implement this short-acting spinal anesthetic to determine the feasibility, practicality, and effectiveness for use for primary THA and TKA surgery, particularly in a tertiary academic institution where surgical duration may exceed that of private practice. This single-center retrospective observational study compares whether the use of chloroprocaine for spinal anesthesia provides a sufficient duration of surgical anesthesia for TKAs and THAs and better recovery profile when compared to bupivacaine in the setting of preoperatively performed regional analgesic blocks for postoperative pain relief.

Parts of this article were previously presented as a meeting abstract at the 2021 American Society of Regional Anesthesia and Pain Medicine (ASRA) Regional Anesthesiology and Acute Pain Medicine Meeting on May 14, 2021.

## Materials and methods

Study design

After receiving IRB approval, we conducted a retrospective review and identified patients by Current Procedural Terminology (CPT) codes who had received spinal anesthesia for primary total knee arthroplasties (TKA) and total hip arthroplasties (THA) at University of Florida Health between 9/1/2019 and 2/21/2020.

Demographic and preoperative data collected included age, body mass index (BMI), sex, patient comorbidities, type of surgery (hip vs knee arthroplasty), peripheral nerve block (PNB) type, and dose and concentration of local anesthetic for PNB. Intraoperative data collected included time of spinal anesthetic administration, intrathecal local anesthetic type, dose, and baricity, duration of surgery, and need for conversion to general anesthesia or escalation of propofol sedation. Duration of surgery was calculated from time of incision to end of intraoperative data collection. These time points were selected because of variability of intraoperative documentation; however, certain time points, including incision time and data collection end time, were required data points in the electronic medical record (EMR) and therefore available for every patient. Conversion to general anesthesia was defined as insertion of supraglottic airway/laryngeal mask airway (LMA) or endotracheal tube (ETT) or propofol infusion running at greater than or equal to 100mg/kg/min in the last 30 minutes of the case.

Postoperative data collected included time from spinal anesthetic placement to spontaneous micturition, time to passing physical therapy’s (PT) post-anesthesia care unit (PACU) ready-for-discharge assessment, time to PACU discharge, and whether PT was completed on the day of surgery. If an inpatient required boarding in the PACU because a hospital room was not available, boarding time was not included in PACU time. Continuous variables were compared using One-Way ANOVA. Means were compared using Tukey’s Honest Significant Difference. Statistically significant differences were defined by p-value < 0.05.

Anesthesia and perioperative care

All patients undergoing TKAs were offered continuous femoral nerve catheters and single injection infiltration between the popliteal artery and capsule of the knee (iPACK) blocks. All patients undergoing THAs were offered continuous femoral nerve catheters with single injection obturator nerve blocks. Peripheral nerve blocks were placed in the preoperative holding area under sedation with midazolam and alfentanil by the acute pain service. The intraoperative anesthesiology team performed the spinal anesthetic in the operating room (OR). Supplemental sedation, usually consisting of a propofol infusion, was provided for patient comfort during the surgery. The type and dosing of the spinal anesthetic and sedation were chosen by the intraoperative anesthesiologist.

## Results

One hundred and twelve patients were identified as having undergone primary THA and TKA under spinal anesthesia within the specified date range. Patients were categorized into three groups based on the type of local anesthetic used for their spinal anesthesia: hyperbaric bupivacaine (HB group) (73 patients), isobaric bupivacaine (IB group) (11 patients), and chloroprocaine (C group) (28 patients).

Patient demographics are listed in Table 1. Intraoperative information, including spinal medication dosing, surgery duration, and conversions to general anesthesia, are in Table 2. Table 3 lists important PACU events including time from spinal to micturition, physical therapy, and PACU discharge.

**Table 1:** Demographics and preoperative metrics (means include standard error) TKA: total knee arthroplasty. THA: total hip arthroplasty

	Chloroprocaine (N = 28)	Hyperbaric bupivacaine (N = 73)	Isobaric bupivacaine (N = 11)	P-values
Age (years) mean	68.9 (1.66)	66.1 (0.988)	67 (2.93)	0.3456
BMI mean	28.7 (1.19)	32.0 (0.707)	32.6 (2.10)	0.4157
Sex: female	18 (64%)	48(65.8%)	5 (45.5%)	
Sex: male	10 (35.7%)	25 (34.2%)	6 (54.5%)	
TKA	20 (71.4%)	40 (54.8%)	4 (36.4%)	
THA	8 (28.6%)	33(45.2%)	7 (63.6%)	

**Table 2:** Intraoperative details SD: standard deviation. GA: general anesthesia. C: chloroprocaine group. IB: isobaric bupivacaine group. HB: hyperbaric bupivacaine group. LMA: supraglottic airway/laryngeal mask airway. ETT: endotracheal tube.

	Chloroprocaine (N = 28)	Hyperbaric bupivacaine (N = 73)	Isobaric bupivacaine (N = 11)	P-values
Mean local anesthetic dose (mg) (SD)	49.8 (0.189)	14.4 (0.246)	13.6 (0.814)	
Mean duration of surgery (minutes) (SD)	89.3 (4.59)	111.3 (2.73)	111.8 (8.10)	0.0002 (C vs HB); 0.0465 (C vs IB); 0.9992 (HB vs IB)
Conversion to GA: LMA or ETT insertion	1 (3.6%)	4 (5.5%)	0 (0%)	1.0000 (LMA vs ETT)
Conversion to GA: Escalation of propofol to >100mcg/kg/min last 30 minutes of case (no invasive airway)	2 (7.1%)	2 (2.7%)	0 (0%)	0.7377

**Table 3:** Postoperative outcomes. All times listed as mean (standard error) PT: physical therapy. C: chloroprocaine group. IB: isobaric bupivacaine group. HB: hyperbaric bupivacaine group.

	Chloroprocaine (N = 28)	Hyperbaric bupivacaine (N = 73)	Isobaric bupivacaine (N = 11)	P-values
Time from spinal to spontaneous micturition (minutes)	304.8 (50.1)	497.5 (29.7)	408.8 (90.3)	0.0037 (C vs HB); 0.5746 (C vs IB); 0.6203 (HB vs IB)
Time from spinal to PACU discharge (minutes)	275.3 (10.5)	317.9 (6.25)	319.0 (18.5)	0.002 (C vs HB); 0.1046 (C vs IB); 0.9984 (HB vs IB)
Time from spinal to PT visit completion (minutes)	257.2 (11.6)	262.4 (7.04)	260.0 (19.8)	0.9296
Did not receive postop PT on day of surgery	2 (7.1%)	8 (11.0%)	0 (0%)	
PT visit limited by residual motor weakness from spinal	0 (0%)	26 (35.6%)	5 (45.5%)	
PT visit limited by other reason than residual motor weakness from spinal	5 (17.9%)	13 (17.8%)	1 (9.1%)	
No PT (other reasons): Case ended late	2 (7.1%)	7 (9.6%)	0 (0%)	
No PT (other reasons): Pain	1 (3.6%)	1 (1.4%)	0 (0%)	
No PT (other reasons): Nausea/Vomiting	2 (7.1%)	3 (4.1%)	0 (0%)	
No PT (other reasons): Hypotension	1 (3.6%)	5 (6.8%)	0 (0%)	
No PT (other reasons): Dizziness	0 (0%)	2 (2.7%)	1 (9.1%)	
No PT (other reasons): Urinary Retention	0 (0%)	1 (1.4%)	0 (0%)	
No PT (other reasons): Surgical extremity weakness	1 (3.6%)	1 (1.4%)	0 (0%)	
No PT (other reasons): Hypoxemia	0 (0%)	1 (1.4%)	0 (0%)	

In the HB group, the most common local anesthetic dose and concentration was 15mg of 0.75% bupivacaine; for the IB group it was 15mg of 0.5% bupivacaine; for the C group it was 50mg of 1% chloroprocaine.

We found a statistically significant reduction of 193 minutes (P = 0.0037) in mean time to micturition in the C group compared to the HB group. We also found a significant reduction of time from spinal to PACU discharge in the C group compared to the HB group of 42.6 minutes (p = 0.002). A higher percentage of patients in the HB (35.6%) and IB groups (45.5%) had their initial postop PT visit limited by residual motor weakness from spinal anesthesia compared to those in the C group (0%). Those in the HB group (11.0%) had a higher rate of not having a day of surgery postop PT visit compared to both the C group (7.1%) and the IB group (0%). The three most common reasons for a patient not having a day of surgery postop PT visit among the three groups were: the patient arriving at PACU after the physical therapists’ shift ended, hypotension, and nausea and vomiting. 

Between the C and IB groups, there was no statistically significant difference in time to micturition (p = 0.575) or time to PACU discharge (p = 0.105). There was also no significant difference in time to PT completion among any of the groups (p = 0.9296).

Two patients in the IB group and one patient in the C group did not receive peripheral nerve blocks. All patients in the HB group received peripheral nerve blocks. The one patient in the C group who did not receive preoperative peripheral nerve blocks required conversion to general anesthesia. Neither of the two patients in the IB group who did not receive peripheral nerve blocks required conversion to general anesthesia or escalation of propofol.

We also found that the mean duration of surgery in the C group was shorter, mean duration in the C group 89.3 (SD 4.59) minutes compared to a mean of 111.3 (SD 2.73) minutes in the IB group and 111.8 (SD 8.10) minutes in the HB group. The length of surgery was statistically significant between both the C and HB groups (p = 0.0002) and the C and IB groups (p = 0.0465).

## Discussion

Using chloroprocaine for spinal anesthesia can provide adequate surgical coverage for THA and TKA of shorter duration (less than 90 minutes) with a low incidence of conversion to general anesthesia. Compared to bupivacaine, chloroprocaine spinal anesthesia has an improved postoperative recovery profile, especially in terms of return of motor function and POUR. Isobaric bupivacaine has a moderately better recovery profile compared to hyperbaric bupivacaine. This is somewhat contrary to published literature suggesting that isobaric bupivacaine has longer duration of motor and sensory blockade [8].

Conversion to general anesthesia can be attributed to two major reasons: incomplete setup of surgical anesthetic block and prolonged surgical time. While, overall, there were very few cases of inadequate onset of spinal anesthesia, the highest incidence was in the chloroprocaine group, followed by the hyperbaric bupivacaine group, and lowest in the isobaric bupivacaine group. There was no clear documentation found of reason for conversion to general anesthesia for any of the patients.

Conversion to general anesthesia with chloroprocaine spinals can be attributed to a slower onset and shorter duration associated with the use of the 50mg dose of chloroprocaine. This is the dose that is available for each chloroprocaine vial, and allows for approximately 75 minutes before motor resolution. Conversion to general anesthesia in any patient receiving spinal anesthesia could be due to inadequate surgical conditions at the initiation of surgery, surgical time exceeding surgical anesthesia time, spinal procedural failure, local anesthetic failure, and patient’s inherent spinal curvature and inadequate patient positioning resulting in a spinal block that is not high enough for surgery. Compared to isobaric local anesthetic, hyperbaric local anesthetic has the benefit of allowing for patient positioning to assist with spread but may settle improperly if patient is not appropriately positioned after injection. According to Ghisi et al., the average onset time for 50mg intrathecal chloroprocaine is 5.3 minutes [9]. This is faster compared to 5.5-12.3 min for hyperbaric bupivacaine and 8-21.54 min for isobaric bupivacaine [10]. However, the authors have observed that, clinically and anecdotally, onset time for spinal chloroprocaine 50mg tends to be slower than stated in these studies, while isobaric bupivacaine appears faster than stated onset times. Time to surgical block onset was not part of the data collected, and requires further investigation.

Baricity may also play a factor. Of the few patients who had conversion to general anesthesia, four underwent THAs and two underwent TKAs (two THAs and one TKA in each of the hyperbaric bupivacaine and chloroprocaine groups). It is possible that 50mg intrathecal chloroprocaine does not always provide a sufficiently dense motor and sensory block to provide satisfactory surgical conditions of a long enough duration for THA. As for hyperbaric bupivacaine, it is possible that due to patient spinal curvature and inadequate initial positioning after injection, the sensory and motor blockade may not reach a sufficient height for surgery.

Preoperative peripheral nerve block placement, particularly use of a continuous femoral catheter not routinely performed at many institutions, likely affected the results. Typically, chloroprocaine spinals are used for short duration surgeries under 40 minutes. Ghisi et al. found that regression of sensory block by two dermatomes with a 50mg dose occurs on average at 41.7 minutes [8]. It is impressive that the patients in the chloroprocaine cohort were able to undergo surgery with a mean surgical time of 89.3 minutes. It is possible that peripheral nerve blockade of the operative extremity prolonged patient tolerance in the concluding minutes of surgery as the sensory and motor blockade from the spinal anesthetic started to recede. The one patient in the chloroprocaine group who did not receive preoperative peripheral nerve blocks required conversion to general anesthesia despite lower than group mean surgical time of 77 minutes. For this patient, there was no documentation of reason for conversion in the intraoperative record, but mismatch of surgical anesthesia time and surgical time is a possibility.

The mean surgical time in the chloroprocaine group was 22 minutes shorter than both bupivacaine groups. This may be due to patient selection, as bupivacaine spinal anesthesia was selected for surgeries expected to be of longer duration. Additionally, surgeons may have operated more quickly, knowing the shorter spinal duration of chloroprocaine. Typically, the intraoperative anesthesiologist would discuss expected surgical time and complexity with the orthopedic surgeon before making decisions about spinal anesthetic dosing.

This data adds to the growing body of data collected on the efficacy and side effects of spinal anesthesia for THA and TKA in this era of enhanced recovery after surgery and same day discharge. Spinal chloroprocaine 50mg may be adequate for shorter duration TKA but may be less optimal for THA. Successful use of intrathecal chloroprocaine 50mg for longer cases (> 1 hour) may partially rely on dense peripheral nerve blockade. It also provides insight into intraoperative success rates with spinal anesthesia at an academic institution where cases may take longer (both because of trainees and case complexity). Increasing the doses of chloroprocaine appears to prolong the duration surgical block, with 60mg lasting 60min to 90 minutes [11]. Since the collection of data for this retrospective review, the authors have begun to use higher doses of chloroprocaine (up to 75mg) or mepivacaine for spinal anesthesia in attempt to speed onset of action and increase duration of anesthesia for TKA and THA. The efficacy of these modifications require further study. Some of the authors also now preferentially perform chloroprocaine spinals at L2-3 for THAs and L5-S1 for TKAs to optimize the levels covered.

Neuraxial anesthesia has been identified as a risk factor for postoperative urinary retention (POUR) after TKA and THA [12]. Intrathecal chloroprocaine seems to result in faster time to micturition compared to IB or HB, which suggests decreased incidence of POUR. While POUR does not absolutely require admission to treat, ambulatory patients benefit from not requiring an indwelling urinary catheter at home or intermittent catheterizations at home. In addition, intrathecal chloroprocaine allows faster sensory and motor blockade regression, allowing for earlier titration of analgesics and less residual weakness during physical therapy sessions.

Herndon et al. also found in their cohort of patients undergoing THA with chloroprocaine vs bupivacaine spinals that the patients who received chloroprocaine spinals averaged shorter PACU lengths of stay and faster discharge times. Compared to our patient group, these patients received a higher dose of chloroprocaine (60 mg), did not receive PNBs, and had shorter median surgery times (68.2 min for chloroprocaine group, 83.6 min for bupivacaine group) [13].

This retrospective study has many limitations. The bupivacaine spinal doses used in these studied patients were high, particularly in the era of ERAS. Decreased doses of bupivacaine has also been shown to improve the recovery profile though they may result in a higher risk of inadequate spinal anesthesia [14-15]. To allow for earlier discharge from the PACU, we do not recommend routinely using these higher doses. Additionally, with a retrospective data collection, the accuracy of the information is limited by the accuracy of EMR documentation by anesthesia, PT, and PACU staff. Furthermore, few patients received isobaric bupivacaine, underpowering the study. This is likely because isobaric bupivacaine was not as easily accessible compared to hyperbaric bupivacaine, which were prepackaged in the spinal kits. Additionally, this study does not evaluate other intermediate acting spinal anesthetics like mepivacaine. However, it is important to note that of all local anesthetics clinically used for spinals, only certain formulations of bupivacaine and chloroprocaine are FDA-approved for intrathecal use in the United States.

PT and PACU discharge times may not have been related to a patient’s medical issues or recovery timeline. The physical therapists did not know which type of local anesthetic was used for the spinal, so they likely visited the patients at their availability according to their standard routine. PACU discharge time was largely dependent on the patient having worked with physical therapy unless the therapists had already left for the day.

Further studies should include prospective randomized controlled trials using these intrathecal local anesthetics in this surgical patient population with defined local anesthetic dosages, intraoperative sedation protocols, and peripheral nerve block protocols to reduce confounding factors and sample size variability.

## Conclusions

Chloroprocaine is a viable spinal anesthetic option for patients undergoing TKAs and THAs, even in an academic setting with longer duration surgical times and higher complexity surgery, particularly with adjunctive preoperatively placed peripheral nerve blocks. Intrathecal chloroprocaine allows for quicker return of motor function and time to micturition compared to bupivacaine spinals. With an improved postoperative recovery profile and adequate surgical conditions, chloroprocaine spinal anesthesia would be a feasible choice for patients planning on same-day discharge after TKA or THA.
